# Genome-Wide Identification and Expression Analysis of the UGlcAE Gene Family in Tomato

**DOI:** 10.3390/ijms19061583

**Published:** 2018-05-27

**Authors:** Xing Ding, Jinhua Li, Yu Pan, Yue Zhang, Lei Ni, Yaling Wang, Xingguo Zhang

**Affiliations:** Key Laboratory of Horticulture Science for Southern Mountainous Regions (Chinese Ministry of Education), College of Horticulture and Landscape Architecture, Southwest University, Chongqing 400715, China; dingdingxing@email.swu.edu.cn (X.D.); ljh502@swu.edu.cn (J.L.); pany1020@swu.edu.cn (Y.P.); zy18109037077@163.com (Y.Z.); m15090062251@163.com (L.N.); yalingwangx@163.com (Y.W.)

**Keywords:** *Solanum lycopersicum*, *UGlcAE* gene family, identification, characterization, plant hormones, gene expression

## Abstract

The UGlcAE has the capability of interconverting UDP-d-galacturonic acid and UDP-d-glucuronic acid, and UDP-d-galacturonic acid is an activated precursor for the synthesis of pectins in plants. In this study, we identified nine *UGlcAE* protein-encoding genes in tomato. The nine *UGlcAE* genes that were distributed on eight chromosomes in tomato, and the corresponding proteins contained one or two trans-membrane domains. The phylogenetic analysis showed that *SlUGlcAE* genes could be divided into seven groups, designated *UGlcAE1* to *UGlcAE6*, of which the *UGlcAE2* were classified into two groups. Expression profile analysis revealed that the *SlUGlcAE* genes display diverse expression patterns in various tomato tissues. Selective pressure analysis indicated that all of the amino acid sites of SlUGlcAE proteins are undergoing purifying selection. Fifteen stress-, hormone-, and development-related elements were identified in the upstream regions (0.5 kb) of these *SlUGlcAE* genes. Furthermore, we investigated the expression patterns of *SlUGlcAE* genes in response to three hormones (indole-3-acetic acid (IAA), gibberellin (GA), and salicylic acid (SA)). We detected firmness, pectin contents, and expression levels of UGlcAE family genes during the development of tomato fruit. Here, we systematically summarize the general characteristics of the *SlUGlcAE* genes in tomato, which could provide a basis for further function studies of tomato *UGlcAE* genes.

## 1. Introduction

As a major component of the primary cell walls of plants [1], pectins are essential for remodeling cell wall and normal cell-cell adhesion during cellular growth [2,3,4,5]. d-galacturonic acid (GalA) is the constituent of the capsular polysaccharides and lipopolysaccharides of several bacterial species [6]. In plants, GalA residues, which are the precursor of pectin formation, are contained in the backbone of all pectin polymers [7]. UDP-d-galacturonic acid (UDP-GalA), which is the activated nucleotide sugar form of GalA, is required in the synthesis of GalA-containing polymers. UDP is the abbreviation of uridine diphosphate and it is a nucleotide diphosphate that is made up of a pyrophosphate group, a pentose ribose, and a nucleated base uracil. UDP-GalA is synthesized via 4-epimerization of UDP-d-glucuronic acid (UDP-GlcA), which is a nucleotide sugar that is formed by the reputed inositol oxygenation pathway [8] or by the dehydrogenation of UDP-d-glucose (UDP-Glc) in the upstream [9]. Therefore, enzymes that are related to the formation of UDP-GalA and UDP-GlcA are likely to play critical roles in pectin biosynthesis [7,10].

UDP-d-glucuronic acid 4-epimerase (UGlcAE) is capable of reversibly converting UDP-GlcA and UDP-GalA [6,11]. According to previous literatures [12,13,14,15], UDP-d-glucuronic acid 4-epimerase is another name of UDP-d-glucuronate 4-epimerase. Both GAE and UGlcAE are the abbreviations of UDP-d-glucuronic acid 4-epimerase. The abbreviation is unified as UGlcAE in this study.

The UGlcAE, which is a specific membrane-bound 4-epimerase [13], is considered to evolve from some chlamydial bacteria [15]. The UGlcAE is also recognized as a key enzyme in regulating pectin biosynthesis due to its function. In 1958, the isolation of the epimerase was firstly reported [16], and subsequently it was also isolated from *Cyanobacterium anabaena* flos-aquae [17,18] and plants [10,19,20,21,22].

Although its function is believed to interconvert UDP-GlcA and UDP-GalA, the UGlcAEs from different organisms have distinct biochemical properties. For example, some UGlcAEs were substrate specific [6,11,12,13,14], while the others displayed substrate promiscuity [23]. In addition, UGlcAEs in *Poaceae* species differed from homologs in *Arabidopsis* [13,14], and different UGlcAEs in the same species also shows biochemical properties that varied differentially. For example, UGlcAE3 in *Ornithogalum caudatum* could catalyse the reversible conversion of UDP-GalA and UDP-GlcA; however, OcUGlcAE1 and OcUGlcAE2 did not have this activity [24].

The evolutionary relationship of the *UGlcAE* gene family in plants is not clear, and we hardly know anything about this gene family in tomato (*Solanum lycopersicum*). Tomato, a berry fruit, is considered to be an important economically vegetable worldwide due to its good quality and high yield [25]. With the completion of the whole genome sequencing of tomato [26], it promotes the genome-wide identification of gene families and functional analysis in tomato [27]. Therefore, studies of the *UGlcAE* gene family in tomato could develop potential strategies for improving *Solanum*-related crops genetically and stimulate new research directions, and considering the potentially important functions of the UGlcAE proteins can expand our knowledge of tomato UGlcAE isoforms. In this study, we identified and characterized the tomato *UGlcAE* gene family on a genome-wide scale, and the tissue- and organ-specific expression of *UGlcAE* family under the normal conditions and in response to three hormone treatments were analyzed according to *cis*-acting elements analysis. Analysis of this family not only identifies its members and characteristics in tomato, but it also lays a foundation for future functional analyses of *UGlcAE* genes.

## 2. Results

### 2.1. The Identification of UGlcAE Gene Family in Tomato

To identify the *UGlcAE* genes in the tomato, we searched for sequences that contained the particular domain in the tomato protein database using the hidden Markov model (HMM) model of PF01370, and we found nine potential genes (Table 1). The open reading frame (ORF) lengths of *UGlcAE* that were identified in this study ranged from 1221 bp to 1359 bp, encoding peptides varied from 406 to 452 amino acids (aa). All nine *UGlcAE* genes had a single exon.

### 2.2. Phylogenetic Analysis of the UGlcAE Genes in Tomato and Other Species

**Figure 1:**
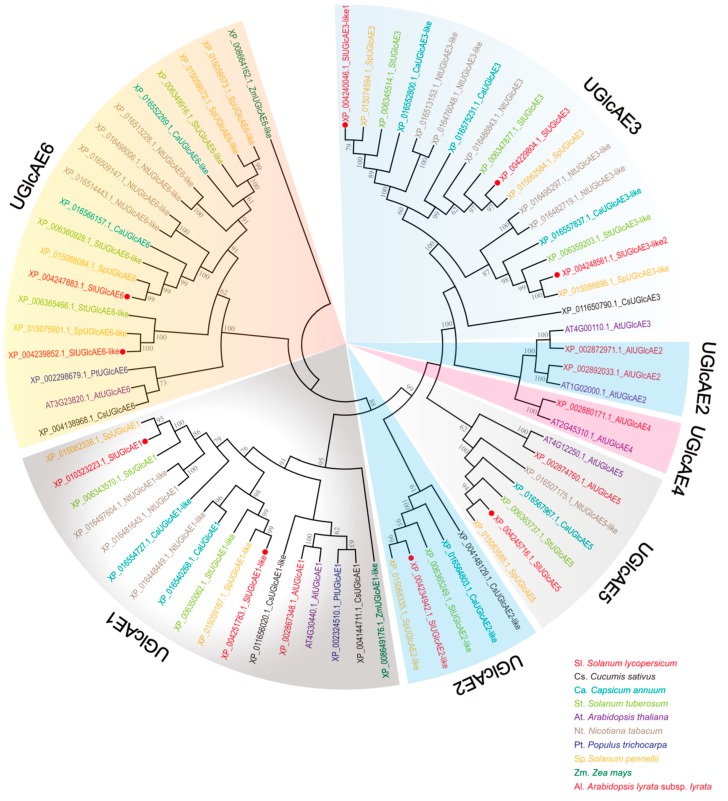
Phylogenetic analysis of the *UGlcAE* gene family based amino acids in tomato and other nine species. The unrooted neighbor-joining phylogenetic tree is generated by MEGA 5. The sequence names included three parts: the source numbers from NCBI, the abbreviation of species names, and their respective subfamilies. Red dots highlight the tomato *UGlcAE* genes.

To evaluate the classification of the *UGlcAE* genes in *S. lycopersicum*, we analyzed the sequence features in 10 different species, including *S. lycopersicum*, *C. sativus*, *C. annuum*, *S. tuberosum*, *A. thaliana*, *N. tabacum*, *P. trichocarpa*, *S. pennelli*, *Z. mays*, and *A. lyrata* subsp. *lyrata*, and we constructed a unrooted phylogenetic tree of the *UGlcAE* genes (Figure 1) using the N-J methods. The orthologous relationships were evident. Only the tree topology is shown, and the branch lengths do not represent the estimated numbers of amino acid replacements [28].

Combing with the sequence similarity of the mature proteins, the employed *UGlcAE* genes are distributed into seven groups (Figure 1). In addition to *UGlcAE2* being divided into two clusters, the other five subfamilies are clustered separately.

Interestingly, the *UGlcAE4* subfamily is specifically present in *A. thaliana* and *A. lyrata* subsp. *lyrata*, whereas it is absent from other species in this study. This implies that *UGlcAE4* may be associated with distinctive functions. It is noteworthy that *UGlcAE2* genes were classified into two different groups based on their evolutionary relationship. This finding indicates that *UGlcAE2* genes may evolve into new features, which have not been known until today. Moreover, phylogenetic analyses showed that the *UGlcAE3* gene in *A. thaliana* was clustered together with *UGlcAE2* genes of *A. thaliana* and *A. lyrata* subsp. *lyrata*, suggesting that there may be some gene fusion among them.

### 2.3. Structures of the UGlcAE Genes in the Tomato and Arabidopsis Thaliana

**Figure 2:**
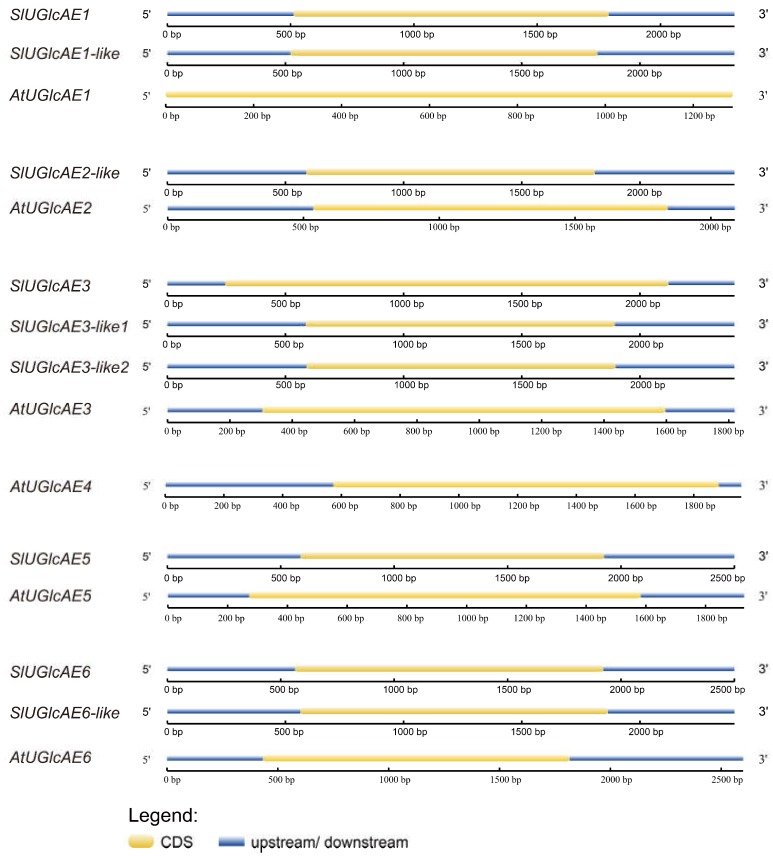
Exon-intron structures of nine tomato *UGlcAE* genes and six *Arabidopsis thaliana UGlcAE* genes. The yellow sections represent the exons, and the blue parts indicate upstream/downstream regions.

Introns, especially UTR introns, in *UGlcAE* genes may influence the expression level [29]. To analyze the structural characteristics of the *UGlcAE* genes in tomato and *Arabidopsis thaliana*, their gene structures were mapped according to the genome sequences and corresponding coding sequences of *SlUGlcAE* and *AtUGlcAE* genes (Figure 2). We found that all of the *SlUGlcAE* and *AtUGlcAE* genes do not contain intron in their genomic sequences. In other words, nine *UGlcAE* genes in the tomato and six *UGlcAE* genes in *Arabidopsis thaliana* are single exon structures.

### 2.4. Chromosomal Distribution of the UGlcAE Genes in Tomato

**Figure 3:**
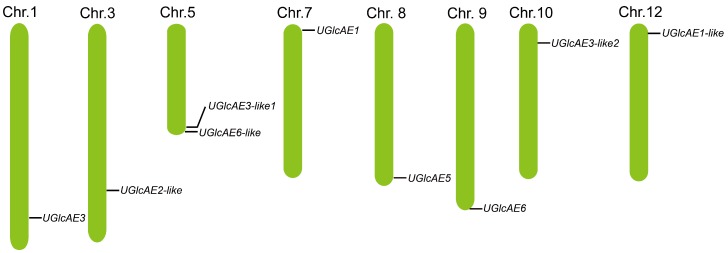
Chromosomal localization of the UGlcAE family genes in tomato. The numbers above each chromosome indicates the chromosome number.

To characterize the distribution of *UGlcAE* genes in the tomato genome, the physical locations of *UGlcAE* genes on the tomato chromosomes were obtained. According to the genomic sequences of *UGlcAE* genes, nine *SlUGlcAE* genes were mapped to eight chromosomes, including chromosome 1, 3, 5, 7, 8, 9, 10, and 12 without regularities of tandem duplication, whose positions were indicated by the black lines in the tomato chromosomes (Figure 3). Two *UGlcAE* genes (*SlUGlcAE3-like1* and *SlUGlcAE6-like*) were located on chromosome 5, and the other seven genes (*SlUGlcAE1*, *SlUGlcAE1-like*, *SlUGlcAE2-like*, *SlUGlcAE3*, *SlUGlcAE3-like2*, *SlUGlcAE5*, and *SlUGlcAE6*) were assigned to different chromosomes, but no gene was mapped to chromosome 2, 4, 6, and 11. There were no tandem duplication events among *UGlcAE* family members of tomato, suggesting that the functional differentiation may exist among the *SlUGlcAE* family members. Almost all of the *UGlcAE* genes in tomato are located near the ends of the chromosome.

### 2.5. Sequence Alignments and Hydrophilicity Analysis of SlUGlcAE Family

**Figure 4:**
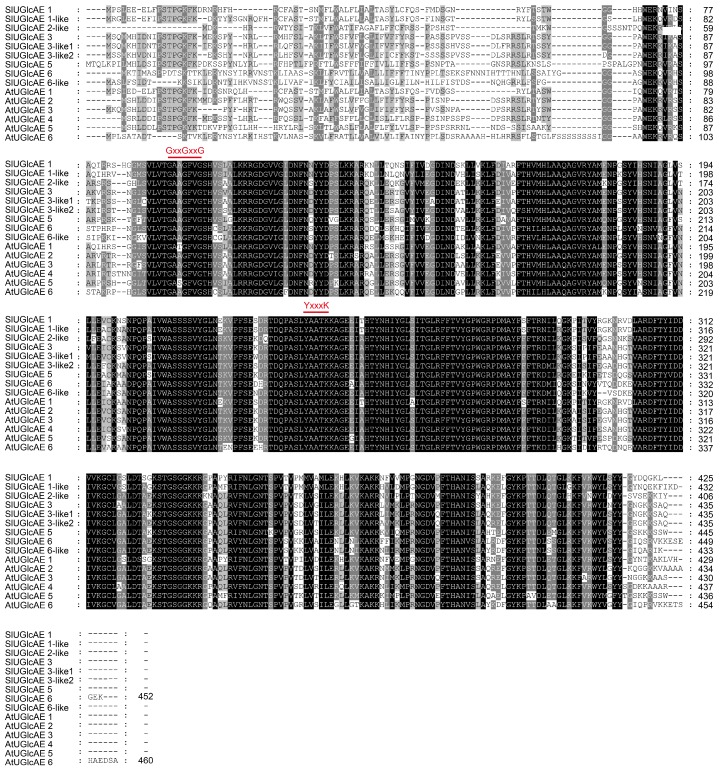
Amino acid sequence alignment of SlUGlcAE in tomato and AtUGlcAE in *Arabidopsis thaliana*. The black backgrounds indicate the strictly conserved residues, and the gray backgrounds indicate the similar amino acid residues. The GxxGxxG and YxxxK motifs are marked above the sequence alignment in red.

UGlcAE is one of the short-chain dehydrogenase/reductase (SDR) enzyme families, and therefore the amino acid sequences of SlUGlcAE and AtUGlcAE contained two conserved motifs that existed in SDR protein families [30,31]. As shown in Figure 4, the two motifs contain an N-terminal GxxGxxG (x represents any amino acid) sequence to bind to NAD (P)^+^, and a motif (YxxxK), which play a catalytic role [32].

As indicated in Supplementary Materials Figure S1, all of the SlUGlcAE proteins were trans-membrane proteins. The result was consistent with previous reports [13,14,24]. Among the nine proteins, most of SlUGlcAEs had only one trans-membrane helice with more than 80% probability, except that SlUGlcAE1 and SlUGlcAE5 were more likely to contain two trans-membrane helices.

### 2.6. Spatiotemporal Expression Patterns Analysis of UGlcAE Genes in Tomato

**Figure 5:**
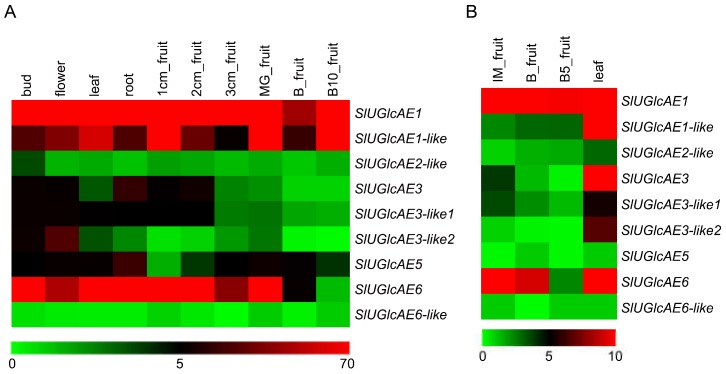
Heatmap analysis of UGlcAE family gene expression in various organs of tomato. (**A**) tomato cultivar *Solanum lycopersicum* (mature green stage (MG), breaker stage (B), ten days after breaker stage (B10)); (**B**) wild relative *Solanum pimpinellifolium* (immature green stage (IMG), breaker stage (B), five days after breaker stage (B5)). The expression data was gained from pubic RNA-seq data and shown as log_2_ as calculated by FPKM values (fragments per kilo base of exon model per million) mapped reads. The green boxes represent the lower expression level, whereas the red boxes represent the higher expression level.

To gain the expression patterns of *UGlcAE* genes in different tissues and organs of tomato, and in the developmental stages of the fruit, we performed expression patterns analysis of the *SlUGlcAE* genes (Figure 5) with the RNA-Seq database on the website of the functional genomics database of the tomato plant. The expression profiles of the nine tomato *UGlcAE* genes showed different patterns of temporal- and tissue-specific expression (Figure 5). The results of the tomato cultivar showed that three genes, including *SlUGlcAE1*, *SlUGlcAE1-like*, and *SlUGlcAE6*, were strongly expressed in the bud, flower, leaf, root, and most fruit ripening stages (Figure 5A). The same three genes (*SlUGlcAE1*, *SlUGlcAE1-like*, *SlUGlcAE6*) showed a similar expression characteristic in the cultivated tomato, with a lower expression at the breaker stage (Figure 5A). In addition, *SlUGlcAE1* and *SlUGlcAE6* had a high expression in leaf and most fruit development stages, and *SlUGlcAE1-like* and *SlUGlcAE3* exhibited a high expression in leaf of currant tomato (Figure 5B). Specifically, *SlUGlcAE6-like* exhibited a very low expression level in root, bud, leave, flower, and fruit of the cultivar tomato and wild relative *Solanum pimpinellifolium* plants (Figure 5A,B).

### 2.7. GO Analysis of the UGlcAE Genes in the Tomato

**Figure 6:**
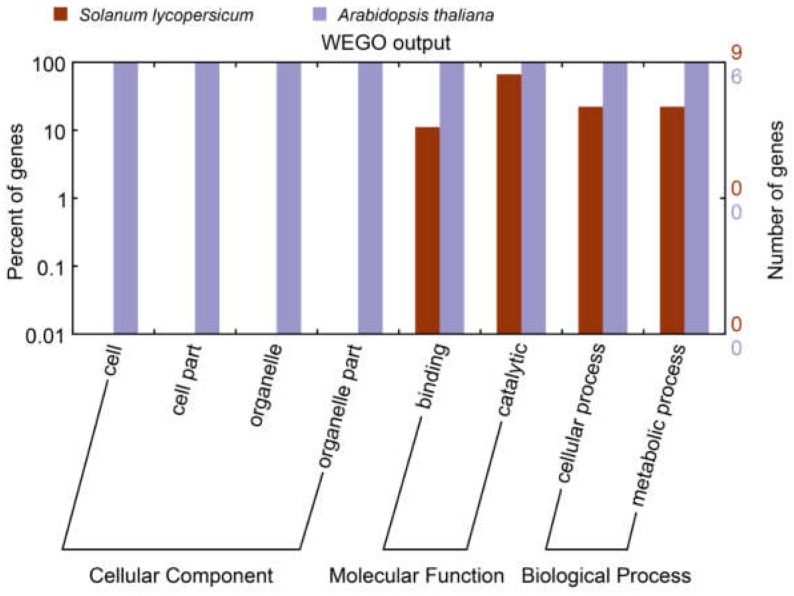
Assignment of Gene Ontology categories to *UGlcAE* genes in tomato. The lengths of the rectangular columns indicate the number of genes that participated in the corresponding classification. The purple rectangular columns mean gene functions of *UGlcAE* in *Arabidopsis*, the red rectangular columns represent gene functions of *UGlcAE* in tomato. All of the gene functions were classified into three categories, which were further divided into eight minor terms.

In order to compare the product functions of *UGlcAE* genes in tomato and *Arabidopsis*, we analyzed *SlUGlcAE* genes and its six orthologous genes in *Arabidopsis*. As it is shown in the gene ontology (GO) map (Figure 6), all of the *UGlcAE* genes of *A. thaliana* are involved in cellular components, molecular functions, and biological processes. However, the *SlUGlcAE* genes just have roles in molecular functions and biological processes.

### 2.8. Selective Pressure on UGlcAE Proteins in the Tomato

**Figure 7:**
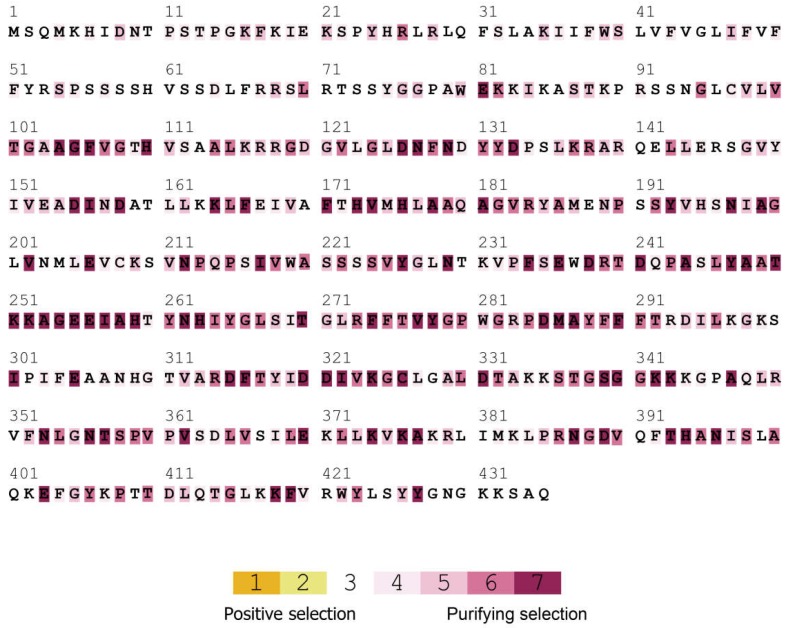
Selection pressure analysis of the UGlcAE proteins in tomato. The red shades represent ω < 1 (purifying selection). Amino acid sequence of SlUGlcAE3-like1 is shown, and the sequences of other SlUGlcAE proteins are presented in the Supplementary Materials Figure S2.

To examine the evolutionary conservation of the UGlcAE proteins, the selective pressure on the UGlcAE was analyzed with SELECTON. We found that the domain of SlUGlcAE3-like1 protein was undergoing strong purifying selection (Figure 7). Selective pressure analyses of the other SlUGlcAE proteins were also analyzed and the results are shown in Supplementary Material Figure S2. These results confirm that the *SlUGlcAE* genes are undergoing strong purifying selection. The amino acids that are emphasized in yellow are under positive selection; however, no positive selection site was found in this selection analysis. These results confirm that these gene family members were very conservative in evolution, which imply them playing a pivotal function in pectin biosynthesis. Selection pressure in the promoter regions of *SlUGlcAE* genes indicated that they are also undergoing negative selection (Figure S3).

### 2.9. Cis-Acting Elements Analysis of the UGlcAE Genes in the Tomato

**Figure 8:**
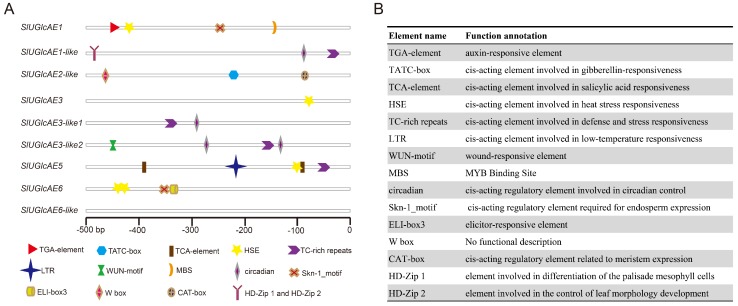
Kinds and numbers of stress-related, hormone-related, and development-related *cis*-elements in the upstream of *SlUGlcAE* genes. (**A**) Various symbols indicate different *cis*-acting elements; and (**B**) Element names and their functional descriptions.

To explore the *cis*-acting elements of *SlUGlcAE* genes, we analyzed the 0.5 kb upstream sequences of nine *SlUGlcAE* genes using online software Plant CARE and the result was shown in Figure 8 and Supplementary Materials Table S1. The analysis result of 1.5 kb upstream genomic sequences of genes was shown in Supplementary Materials Table S2.

Kinds, numbers, and locations of *cis*-elements in the upstream of *SlUGlcAE* genes were shown in Figure 8A, and the functional descriptions of these stress-related, hormone-related, and development-related *cis*-elements were exhibited in Figure 8B. As shown in Figure 8A, there are three *cis*-acting elements that are related to hormone, including TGA-element, TATC-box and TCA-element, and 5 stress-related elements including HSE (heat stress-related element), TC-rich repeats (*cis*-acting element involved in defense and stress responsiveness), LTR (*cis*-acting element involved in low-temperature responsiveness), WUN-motif (wound-responsive element), and MBS (MYB Binding Site), and seven elements that are involved in development (Skn-1_motif, HD-Zip 1, HD-Zip 2, circadian, CAT-box, W box, and ELI-box3). Among them, the 0.5 kb upstream regions of four *SlUGlcAE* genes were found to be the presence of heat stress-related element (HSE), of which had two HSE elements in the 0.5 kb upstream region of *SlUGlcAE6* and 1 HSE elements in the 0.5 kb upstream regions of *SlUGlcAE1*, *SlUGlcAE3,* and *SlUGlcAE5*. Furthermore, defense- and stress-response element (TC-rich repeats) was identified in the 0.5 kb upstream regions of four *SlUGlcAE* genes (*SlUGlcAE1-like*, *SlUGlcAE3-like1*, *SlUGlcAE3-like2*, and *SlUGlcAE5*), and circadian element (circadian) was found in the 0.5 kb upstream regions of three *SlUGlcAE* genes (*SlUGlcAE1-like*, *SlUGlcAE3-like1,* and *SlUGlcAE3-like2*), and endosperm expression-related element (Skn-1_motif) was discovered in the 0.5 kb upstream regions of two *SlUGlcAE* genes (*SlUGlcAE1* and *SlUGlcAE6*), and other 11 elements are all present in the 0.5 kb upstream regions of only one *SlUGlcAE* gene. Four elements are located in 0.5 kb upstream region of *SlUGlcAE1*, and element numbers are diversified in the 0.5 kb upstream region of other genes, respectively (four in *SlUGlcAE1-like*, three in *SlUGlcAE2-like*, one in *SlUGlcAE3*, two in *SlUGlcAE3-like1*, four in *SlUGlcAE3-like2*, five in *SlUGlcAE5*, four in *SlUGlcAE6*, and zero in *SlUGlcAE6-like*).

No of stress-, hormone-, and development-related element was found in the 0.5 kb upstream region of *SlUGlcAE6-like* gene, and the previous spatial expression patterns showed *SlUGlcAE6-like* was lowly expressed at every stages, which are highly consistent. Thus, we infer that *SlUGlcAE6-like* may not be involved in the process of growth and development. In addition, HD-Zip 1 and HD-Zip 2 are located in the same position of *SlUGlcAE1* and *SlUGlcAE1-like*, implying that the two elements might be closely related and complementary to each other. Furthermore, other stress-, hormone-, and development-unrelated *cis*-acting elements have also been identified. For example, core promoter element (TATA-box) and common *cis*-acting element (CAAT-box) are present in the 0.5 kb upstream regions of all nine *UGlcAE* genes. Light responsive *cis*-acting regulatory elements (GATA-motif, chs-CMA1a, G-box) and enhancer (TA-rich region) could also be found.

### 2.10. Expression Patterns of SlUGlcAE Family Genes in Response to IAA, GA and SA

**Figure 9:**
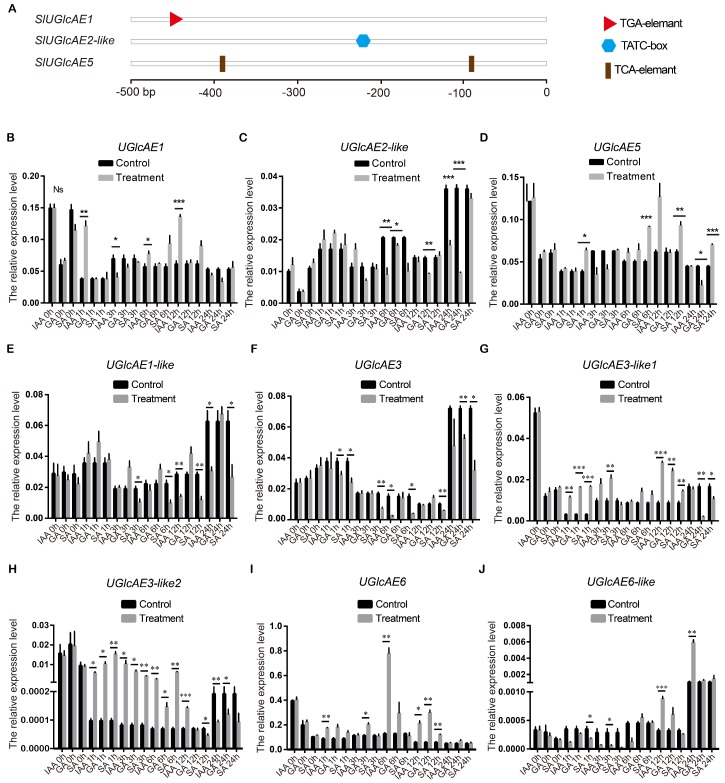
The detection of expressional level of *SlUGlcAE* genes after plant hormone treatments. (**A**) Hormone-related *cis*-elements prediction in the upstream of three *SlUGlcAE* genes (TGA-element: auxin-responsive element, TATC-box: gibberellin-responsive element, TCA-element: salicylic acid-responsive element); (**B**) The qPCR expression analysis of *SlUGlcAE1* response to plant hormone treatments; (**C**) Responses of *SlUGlcAE2-like* to plant hormones; (**D**) Expression profile analysis of *SlUGlcAE5* under plant hormone treatments; (**E**) Expression of *SlUGlcAE1-like* after hormone treatments; (**F**) Expression level of *SlUGlcAE3* after three hormones treatments; (G) Responses of *SlUGlcAE3-like1* under hormone treatments; (**H**) Expression profile analysis of *SlUGlcAE3-like2* to plant hormones; (**I**) The expression analysis of *SlUGlcAE6* under three hormones treatments; and, (**J**) Expression pattern of *SlUGlcAE6-like* response to plant hormone treatments. The error bars represent the SEM. * *p* < 0.05, ** *p* < 0.01, *** *p* < 0.001, Ns: No significant.

Plant hormones, such as IAA, GA, and SA are used as endogenous messengers in response to biotic and abiotic stresses in plants [33]. It has been reported that the treatments of plants by exogenous hormones often lead to transient and rapid transcriptional changes in the whole genome [34]. According to *cis*-acting elements analysis of the *SlUGlcAE* genes upstream, three *cis*-acting elements that are related to plant hormones (IAA, GA, and SA) are located in 0.5 kb upstream genomic sequences of *SlUGlcAE1*, *SlUGlcAE2-like*, and *SlUGlcAE5*, respectively (Figure 9A). Thus, we investigated the expression profiles of *SlUGlcAE1*, *SlUGlcAE2-like,* and *SlUGlcAE5* with IAA, GA, and SA treatments (Figure 9B–D).

The expression of *SlUGlcAE1* in response to IAA was increased at 1 h, decreased at 3 h, and increased thereafter, including 6 and 12 h, and returned to background level until 24 h (Figure 9B). On the whole, the expressional level of *SlUGlcAE1* gene was up-regulated after IAA treatment, although accompanying an unclear reason of being down-regulated only at 3 h. The expressional level of *SlUGlcAE1* gene was not significant different when comparing to the control in response to GA and SA throughout the treated process (Figure 9B). Combining to the result analysis of *cis*-acting elements in *SlUGlcAE1* gene upstream, we found that *SlUGlcAE1* expressional regulation was consistent with TGA *cis*-acting elements existing in the upstream of this gene, which is an auxin-responsive element, while GA or SA responsive element is non-existent (Figure 9A). This indicated that the expression of *SlUGlcAE1* gene could be regulated by IAA, but it could not be regulated by GA and SA (Figure 9B). The *SlUGlcAE2-like* gene under IAA treatment was down-regulated at 6 and 24 h, and no obvious difference was found at other time points (Figure 9C). The change of *SlUGlcAE2-like* expression pattern was unobvious under SA treatment. *SlUGlcAE2-like* showed increasingly strong down-regulation in expression at 6, 12 and 24 h, while similar expression level to the control within the first 3 h under GA treatment (Figure 9C). These results indicated that the expression of *SlUGlcAE2-like* gene could be regulated by GA, and be regulated by IAA irregularly, but not be regulated by SA (Figure 9C). This is aligned with the previous *cis*-acting elements analysis result. That is the fact that there are a gibberellin-responsive element (TATC-box) within 0.5 kb genomic sequences of *SlUGlcAE2-like* gene upstream (Figure 9A) and another gibberellin-responsive element (GARE-motif) within 1.5 kb genomic sequences outside 0.5 kb genomic sequences of *SlUGlcAE2-like* gene upstream (Table S2). There is an auxin-responsive element (AuxRR-core) within 1.5 kb genomic sequences outside 0.5 kb genomic sequences of *SlUGlcAE2-like* gene upstream (Table S2). *SlUGlcAE5* after IAA treatment showed a similar expression level to the control at different time points (Figure 9D). The expression level of *SlUGlcAE5* after GA treatment was no significant change in the first 12 h, but it reduced at 24 h with unknown reasons). *SlUGlcAE5* was up-regulated at 1 h, not affected at 3 h, and up-regulated continuously at later time points, including 6, 12, and 24 h after SA treatment (Figure 9D). The results suggested the expression of *SlUGlcAE5* gene could indeed be regulated by SA. It keeps consistent with the result of *cis*-acting elements analysis, which has a salicylic acid-responsive element (TCA element) in 0.5 kb genomic sequences of *SlUGlcAE5* gene upstream (Figure 9A). Taken together, these data suggest that although the result of *cis*-acting elements analysis may contain a few false positives, the prediction of these three hormone response sites in our study is still relatively reliable.

In addition, we also examined the expression level of the other six *UGlcAE* genes after three hormones treatments. The results were shown in Figure 9E–J. Both *SlUGlcAE1-like* and *SlUGlcAE3* were down-regulated by SA (Figure 9E,F). *SlUGlcAE3-like2* was up-regulated by the three hormones within 12 h of hormone treatment. However, it was down-regulated by three hormones at 24 h (Figure 9H). *SlUGlcAE3-like1*, *SlUGlcAE6*, and *SlUGlcAE6-like* were affected by different hormones at different times after hormones treatments (Figure 9G,I,J).

### 2.11. The Firmness, Pectin Content and the Expression Level of UGlcAE Family Genes in Tomato Fruits at Different Development Stages

**Figure 10:**
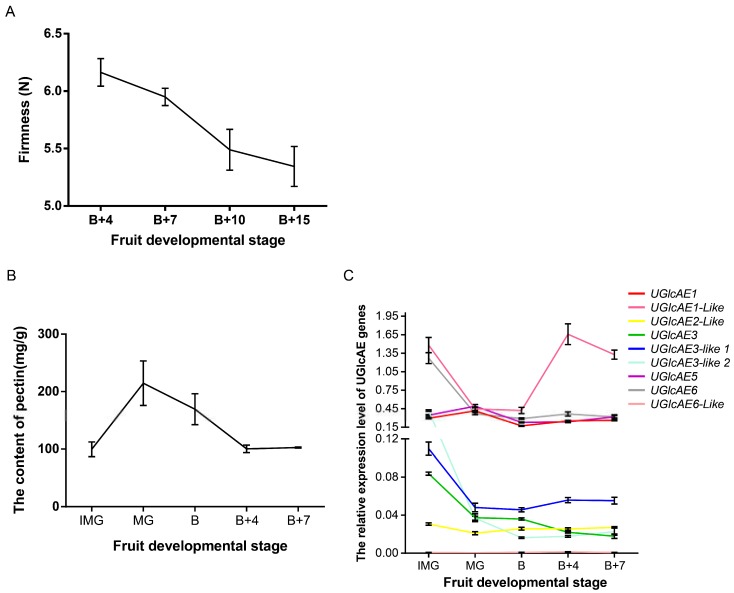
Firmness, pectin contents, and expression levels of UGlcAE family genes during the development of tomato fruit. (**A**) Firmness of tomato fruit in five different stages; (**B**) water-soluble pectin (WSP) contents of fruit in five development stages; and (**C**) Expression level of nine UGlcAE family genes in stages.

To further explore the change of firmness, pectin contents, and expression levels of *UGlcAE* genes, we investigated the firmness, pectin contents, and the expression levels of *UGlcAE* family genes in fruits at different stages of tomato development (Figure 10). As the fruit matured, the firmness of tomato fruit decreased gradually (Figure 10A). The content of water-soluble pectin (WSP) in the development of tomato fruit showed an increased trend, reached a maximum at the MG stage, and then decreased (Figure 10B). The increase of WSP content in the early may be because of the accumulation of pectin as the fruit grows. The content of WSP gradually decreased after the MG stage, probably due to the gradual degradation of partially WSP by some pectinases. As shown in Figure 10C, nine genes of the UGlcAE family have different expression patterns in the development of tomato fruit. Among them, four genes (*UGlcAE1*, *UGlcAE1-like*, *UGlcAE5*, and *UGlcAE6*) showed relatively high expression levels, and other five genes had lower expression levels. The expression levels of both *UGlcAE1* and *UGlcAE5* first increased, reached the maximum value at the MG stage, and then decreased. This is consistent with the trend of WSP content in tomato fruit development.

## 3. Discussion

UGlcAE is capable of reversibly interconverting UDP-GlcA and UDP-GalA, which plays an important role in pectin synthesis. It bring many new opportunities to study gene families in an evolutionary context with various plant genomes being sequenced [4]. To investigate the phylogenetic relationship of *UGlcAE* gene family members, we searched and collected the amino acid sequences of UGlcAE from 10 plant species. Allof the six subfamilies exist in *Arabidopsis thaliana*. However, in other plant species, the numbers of subfamilies of *UGlcAE* genes vary from three to five. Interestingly, the UGlcAE4 subfamily is specifically present in *Arabidopsis thaliana* and *Arabidopsis lyrata* subsp. *lyrata*, whereas it is absent from the other eight plant species. According to this analysis, we infer that the UGlcAE4 protein may have played specific roles in *Arabidopsis*.

It is mentionable that the members of the UGlcAE2 subfamily were not classified into the same cluster. This indicates that there are great differences in the sequences of different members within the UGlcAE2 subfamily. We further speculate that the UGlcAE2 subfamily may be dividing new functions. Moreover, some members of the UGlcAE2 subfamily grouped with UGlcAE3 in *Arabidopsis*. It indicates that there are similarities in the sequences of these UGlcAE2 members and AtUGlcAE3. Therefore, it is likely that the members of the UGlcAE2 subfamily and AtUGlcAE3 might have similar functions or undergo gene fusion.

Similar to the previous studies of the UGlcAE in *Arabidopsis* [12,13], two branches of the phylogenetic tree are trustworthily occupied by UGlcAE1 and UGlcAE6, respectively, meanwhile, UGlcAE2, UGlcAE3, UGlcAE4, and UGlcAE5 are located together in one branch of the phylogenetic tree. This result implies a more ancient role of UGlcAE1 and UGlcAE6, concurrently, the other UGlcAEs might have evolved later [12,13].

In all ten plant species, only *Arabidopsis* contains all of the six *UGlcAE* subfamilies and every subfamily has at least one member. This is comprehensive and regular, which is very congruent with its identity of the model plant.

The expression patterns of the nine genes differed in the different tissues and development stages of tomato. However, it is still possible to find a certain rule from Figure 5. As mentioned by Mølhøj in 2004 [13], the heatmap representation of all the expression patterns reveals that *UGlcAE1* and *UGlcAE6* subfamilies (except *UGlcAE6-like*) were strongly expressed in cultivar tomato, whereas *UGlcAE2*, *UGlcAE3*, and *UGlcAE5* subfamilies were lowly expressed isoforms. However, *UGlcAE6-like* showed considerably lower expression levels in tomato. This is consistent with the result of *cis*-element analysis of *UGlcAE* gene families in Figure 8, which is no significant (stress-, hormone- and development-related) *cis*-acting elements being found within the range of 0.5 kb in front of the *UGlcAE6-like* gene coding region.

The expression trends of *UGlcAE1* and *UGlcAE5* in tomato fruit development were consistent with those of WSP content, indicating that *UGlcAE1* and *UGlcAE5* may be more closely related to the formation of WSP during the fruit ripening when compared to other members of the *UGlcAE* gene family. The expression level of *UGlcAE5* was high in Figure 10C and low in Figure 5A, which indicate that *UGlcAE5* may be easily affected by some factors in the environment and cause its expression level to be unstable. In addition, other results of Figure 10C (high expression level of the three genes (*UGlcAE1*, *UGlcAE1-like*, and *UGlcAE6*) and low expression level of the five genes (*UGlcAE2-like*, *UGlcAE3*, *UGlcAE3-like1*, *UGlcAE3-like2* and *UGlcAE6-like*)) were basically consistent with the results of Figure 5A. This may suggest that the expression of these eight genes is relatively stable during tomato fruit development.

After three hormones treatments, the expression of *UGlcAE1* was more susceptible to IAA, and the expression of *UGlcAE5* was more susceptible to SA. These results suggest that the WSP content of tomato may be more susceptible to IAA and SA in fruit development. *UGlcAE6-like* exhibited the very low expressions in Figure 5, Figure 9J and Figure 10C, indicating that *UGlcAE6-like* is less likely to affect WSP content during the tomato fruit ripening.

Pectin degradation is a major effect on fruit softening [35]. The identifications of the family genes help to understand more about these genes and can better investigate the mechanisms of pectin production and degradation. An in-depth understanding of specific gene expression during ripening and maturation of tomato fruits [36] will enable the precise manipulation of expression of new associate genes to more precisely control the mechanisms of cell wall modification and softening. This is still an outstanding question so far [35].

## 4. Materials and Methods

### 4.1. Data Set Collection and Identification of SlUGlcAE Genes

The protein databases of all ten species were retrieved from the National Center for Biotechnology Information (NCBI) FTP site (available online: http://www.ncbi.nlm.nih.gov/Ftp/). The cDNA, CDS, and genome sequence data in tomato were downloaded from the Solanaceae Genomics Network (SGN) (available online: http://solgenomics.net) [37] and Tomato Functional Genomics Database (TFGD) (available online: http://ted.bti.cornell.edu) [38]. Other information and sequences of *Arabidopsis thaliana* UGlcAEs (AtUGlcAEs) were obtained from the *Arabidopsis* Information Resource (TAIR; available online: http://www.arabidopsis.org/) [39]. The UGlcAE proteins of tomato (SlUGlcAEs) were predicted depending on the UGlcAE hidden Markov model (HMM) profile from the Pfam database (available online: http://pfam.sanger.ac.uk/) [40], which was used to search the *S. lycopersicum* UGlcAE proteins sequences by the HMMSEARCH program from HMMER software (available online: http://hmmer.janelia.org) [41]. In the case of the uncompleted protein databases, all of the results were then used as queries in TBLASTN searches against the tomato genomic sequences. To further confirm UGlcAE proteins, the domains of candidate sequences were predicted with the Pfam online server (available online: http://pfam.sanger.ac.uk/) [40] and SMART online server (available online: http://smart.embl-heidelberg.de/) [42]. The tomato genomic sequences were also checked using BLASTP at the NCBI site (available online: http://blast.ncbi.nlm.nih.gov), retaining only those sequences with highly significant matches to annotated UGlcAE proteins. The same procedure was used to search UGlcAE family members in the protein databases of the following nine species: *Cucumis sativus*, *Capsicum annuum*, *Solanum tuberosum*, *Arabidopsis thaliana*, *Nicotiana tabacum*, *Populus trichocarpa*, *Solanum pennelli*, *Zea mays*, and *Arabidopsis lyrata* subsp. *lyrata*.

The tomato *UGlcAE* gene subfamilies were named according to the orthologous *UGlcAE* genes in the *A. thaliana* genome. The subfamilies of *UGlcAE* genes in the tomato were distinguished by Arabic numerals, and different members of a subfamily were designated with the numbers.

### 4.2. Phylogenetic Analysis

A phylogenetic tree of UGlcAE was constructed by analyzing full-length proteins from *S. lycopersicum*, *C. sativus*, *C. annuum*, *S. tuberosum*, *A. thaliana*, *N. tabacum*, *P. trichocarpa*, *S. pennelli*, *Z. mays*, and *A. lyrata* subsp. *lyrata* in the MEGA5 software (Center for Evolutionary Medicine and Informatics, Arizona State University, Tempe, AZ, USA) using the Neighbor-Joining method [43]. Bootstrap analysis was employed using 1000 replicates.

### 4.3. Selective Pressure Analysis on UGlcAE Proteins in the Tomato

The ratio of non-synonymous to synonymous substitutions (dN/dS; termed ω) at each codon site of each protein was identified, according to an empirical Bayesian method using the Server for the identification of site-specific positive selection and purifying selection (SELECTON version 2.4, Tel Aviv University, Tel Aviv, Israel [44,45]. Selection pressure analysis can be used to identify purifying or positive selection of specific areas in a sequence, and the sites that ω values significantly >1 or <1 suggest positive (Darwinian) or purifying, respectively [46]. The selection pressure acting on the coding sequences of the *SlUGlcAE* genes was recognized with the M8 model (extra category ωs ≥ 1, beta distribution, and positive selection allowed). In order to ensure the accuracy of the results, a likelihood ratio test was used to test the significance of the ω values [47], which compares two nested models: a null model that assumes no positive selection (M8a) and an alternative model that assumes positive selection (M8). Non-nested models, including M8a (extra category ωs set to 1) and MEC (positive selection allowing model), were also used in the pressure analysis.

### 4.4. Chromosomal Location

Locations of the *UGlcAE* genes on the tomato chromosomes were obtained using NCBI website (available online: http://www.ncbi.nlm.nih.gov/mapview/), according to their positions in the SGN (available online: http://solgenomics.net/) [37].

### 4.5. Gene Structure Analysis

To analyze gene structure, the exon, and intron structures of *SlUGlcAE* and *AtUGlcAE* genes were generated using the Gene Structure Display Server 2.0 (available online: http://gsds.cbi.pku.edu.cn) [48] by aligning the CDS sequences with the corresponding genomic DNA sequences from the SGN (available online: http://solgenomics.net/) [37].

### 4.6. Gene Ontology Analysis

For the gene ontology (GO) analysis, the *UGlcAE* gene family members in tomato and *Arabidopsis* were classified according to their each GO numbers from the SGN (available online: http://solgenomics.net/) [37] and the TAIR (available online: http://www.arabidopsis.org/) [39]. After being normalized by the online service CapitalBio Molecule Annotation System (MAS) 3.0 (available online: http://bioinfo.capitalbio.com/mas3/) [49], their GO numbers were identified and visualized with BGI WEGO (available online: http://wego.genomics.org.cn/cgi-bin/wego/index.pl) [50].

### 4.7. Analysis of Expression Profile of UGlcAE Genes in Tomato Various Tissues

The expression profile was obtained through analyzing microarray data. The microarray data were downloaded from the Tomato Functional Genomics Database (available online: http://ted.bti.cornell.edu/cgi-bin/TFGD/digital/home.cgi) [38], including the *UGlcAE* genes expression in 10 tissues (bud, flower, leaf, root, 1 cm fruit, 2 cm fruit, 3 cm fruit, mature green stage (MG) fruit, breaker stage (B) fruit, and ten days after breaker stage (B10) fruit) of the tomato cultivar (*Solanum lycopersicum*), and four tissues (immature green stage (IMG) fruit, breaker stage (B) fruit, five days after breaker stage (B5) fruit, and leaf) of the wild species (*Solanum pimpinellifolium*). Only genes with an at least five units average expression signal at one time point and the similar trend in different biological replicates were considered to be expressed at the time point. The expression patterns of the *SlUGlcAE* genes were estimated by intensity values and were visualized using MultiExperiment Viewer (Broad Institute of MIT and Harvard University, Boston, MA, USA [51].

### 4.8. Sequence Alignments and Prediction of Transmembrane Domains of SlUGlcAE Family

All nine SlUGlcAE and six AtUGlcAE protein sequences were aligned using the (version 5.0.6, North Carolina State University, Raleigh, NC, USA), and then the results were output by genedoc program. Next, the hydrophilicity of the SlUGlcAE protein sequences was predicted by the trans-membrane Hidden Markov model algorithm (available online: http://www.cbs.dtu.dk/services/TMHMM/) [52].

### 4.9. The Analysis of SlUGlcAE Family Protein Domains

The domains of UGlcAE family proteins in tomato were analyzed by the Pfam (available online: http://pfam.xfam.org/search) [40].

### 4.10. Cis-Elements in the Upstream of SlUGlcAE Genes

For identifying the *cis*-acting elements of UGlcAE genes upstream, we obtained the sequences of upstream regions (1.5 kb) of nine *SlUGlcAE* genes from NCBI (available online: https://www.ncbi.nlm.nih.gov/) and identified *cis*-acting motifs by PlantCARE (available online: http://bioinformatics.psb.ugent.be/webtools/plantcare/html/) [53].

### 4.11. Hormone Treatments

*S. lycopersicum* plants were grown at 25 ± 2 °C with a 12 h light/dark photoperiod. The humidity was maintained at approximately 60% to 70%, and the photosynthetic photon flux density was controlled at about 120 μmol photons/m^2^/s. When the seedlings were six weeks old, the plants were treated with IAA (100 μM), GA (100 μM) and SA (100 μM), respectively [54]. Plant leaves were collected at 0, 1, 3, 6, 12, and 24 h after treatments, immediately frozen in liquid nitrogen, and then stored at −80 °C until use.

### 4.12. Plant Materials

Other *S. lycopersicum* seedlings were grown in the same conditions with the above mentioned seeds (see Section 4.11). Different fruits were harvested in the following five stages: immature green stage (IMG), mature green stage (MG), breaker stage (B), four days after breaker stage (B + 4), and seven days after breaker stage (B + 7). All of the plant samples were retrieved at the same time each day, and then frozen in liquid nitrogen and stored at −80 °C.

### 4.13. Real-Time PCR

Total RNA was extracted from the leaves using the Total RNA Kit (BioTeke Corporation, Beijing, China), following the manufacturer’s instructions. Integrity of the RNA was verified by agarose gel electrophoresis. Synthesis of the cDNA was performed from the total RNA samples using the PrimeScript™ RT Reagent Kit, according to the protocol with gDNA Eraser (TaKaRa, Dalian, China). All of the primer sequences are shown in Table 2. *EF1α* gene was used as the internal control under abiotic stress [55], and the *SlCAC* gene was selected as an internal standard during tomato development [56] to quantitate the expression of *SlUGlcAE* genes. Real-time PCR was performed using CFX96 Touch™ real-time PCR system (Bio-Rad, Hercules, CA, USA) with a SYBR Premix Ex Taq™ II Kit (Bio-Rad). The reactions were carried out in the following conditions: denaturation at 94 °C for 4 min, 40 cycles of 5 s at 95 °C, 30 s at 60 °C, 15 s at 95 °C, 20 s at 60 °C, and 15 s at 95 °C. Three biological duplications were used. The 2^−∆∆*C*t^ method was used to visualize and analyze the real-time PCR data [57,58].

### 4.14. Fruit Firmness Measurement and Determination of Water-Soluble Pectin Content

As described by Wu and Abbott [59], the fruit firmness was quantified using a Firmness tester (GY-2). Fifteen unbroken tomatoes were taken from each group. The equator of the fruit was placed under a flat probe, and the maximum value was read after pressing down. Each fruit was measured at least three times. Test parameters: Probe pressure rate 1 mm/s, Pressing distance 3 mm.

The content of pectin in tomato fruit was detected by the water-soluble pectin content kit of Suzhou Keming Biotechnology Co., Ltd. (Suzhou, China The principle of determination is to use the acid solution to extract water-soluble pectin, and to determine the content of pectin by carbazole colorimetry. Pectin is hydrolyzed to galacturonic acid, which condenses with carbazole reagent in sulfuric acid solution. The resulting material has a maximum absorption peak at 530 nm.

## 5. Conclusions

The *Solanaceae* genus is one of the most morphologically various plant families, with more than 3000 described species being distributed worldwide [60]. Apart from being of economic value, tomato is also a model crop for fleshy fruit development [35,61]. In addition, tomato is still the first horticultural crop for which its genome has been sequenced [62]. In this study, we identified nine *SlUGlcAE* genes and analyzed the spatiotemporal expression patterns, the phylogenetic relationships, the selective pressure, the *cis*-acting elements, and so on. We also focused on the response patterns of nine *SlUGlcAE* genes to IAA, GA, and SA, according to the results of *cis*-acting elements analysis. Moreover, the firmness decreased gradually, and WSP showed an increased trend, reached a maximum at the MG stage, and then decreased in the development of tomato fruit. All of the results above have allowed for us to identify tomato orthologs that are related to known *UGlcAE* genes in *Arabidopsis* for in-depth studies. It would also accelerate for executing functional studies based genomics to elucidate their elaborate roles in tomato fruit development, and to be helpful for revealing the roles of other members in the *Solanaceae* genus.

## Figures and Tables

**Table 1:** Information about the nine isoforms of the tomato SlUGlcAE gene family.

No.	Gene Accession No.	NCBI Name	Chr.	Gene Name	Location	*Arabidopsis* Homologous	Size (AA)	ORF (bp)	Exon
1	Solyc07g006220	XP_010323223	7	SlUGlcAE1	ch07:1039601-1041900	AT4G30440	425	1278	1
2	Solyc12g010540	XP_004251783	12	SlUGlcAE1-like	ch12:3531001-3533400	AT4G30440	432	1299	1
3	Solyc03g083550	XP_004234942	3	SlUGlcAE2-like	ch03:53489301-53491700	AT1G02000	406	1221	1
4	Solyc01g091200	XP_004229804	1	SlUGlcAE3	ch01:84887601-84890000	AT4G00110	435	1308	1
5	Solyc05g050990	XP_004240046	5	SlUGlcAE3-like1	ch05:61200401-61202800	AT4G00110	435	1308	1
6	Solyc10g018260	XP_004248561	10	SlUGlcAE3-like2	ch10:7314201-7316600	AT4G00110	435	1308	1
7	Solyc08g079440	XP_004245716	8	SlUGlcAE5	ch08:62963401-62965900	AT4G12250	445	1338	1
8	Solyc09g092330	XP_004247883	9	SlUGlcAE6	ch09:71458501-71461000	AT3G23820	452	1359	1
9	Solyc05g053790	XP_004239852	5	SlUGlcAE6-like	ch05:63814001-63816400	AT3G23820	433	1302	1

**Table 2:** Primer sequences used for quantitative real-time PCR in the paper.

Primer Name	Sense Sequence (5′ → 3′)	Antisense Sequence (5′ → 3′)
*EF1a*	TACTGGTGGTTTTGAAGCTG	AACTTCCTTCACGATTTCATCATA
*CAC*	CCTCCGTTGTGATGTAACTGG	ATTGGTGGAAAGTAACATCATCG
*SlUGlcAE1*	TGTAAAATGGCTAATCCACAACCT	AAAAACCGCAATCCAGTAATCG
*SlUGlcAE1-like*	ACCGGTGTTTCGCTTCAACGAGT	AAGACTACCCCATGTGGAGGAGAG
*SlUGlcAE2-like*	GCGAGTCTATACGCTGCCACA	CGTCTTCTTACCACCACTTCCTG
*SlUGlcAE3*	CAACCCCAGGAAAGTTCAAGATGG	GACGAAGAAGCTGGAGATCTGTAG
*SlUGlcAE3-like1*	AGGCAGCTAATCATGGCACAGTC	AAGATCAGATACCGGGACAGGTG
*SlUGlcAE3-like2*	TCATGGGACTGTTGCTAGGGACT	CCTTGGCAACTTCATCACAGCTC
*SlUGlcAE5*	TGTAAAATGGCTAATCCACAACCT	AAAACCGCAATCCAGTAATCG
*SlUGlcAE6*	CCACCTGACACAAGCAAAACCAC	GGAGGATAGAAGGTTATGGGTAGTGG
*SlUGlcAE6-like*	GGACTGATCAACCAGCTAGTCTC	CGTAAACCTTGATCGGCTTCCCTTG

## Supplementary Materials

Supplementary materials can be found at http://www.mdpi.com/1422-0067/19/6/1583/s1.

## Abbreviations

UGlcAE/GAE	UDP-d-glucuronic acid 4-epimerase
IAA	indole-3-acetic acid
GA	gibberellin
SA	salicylic acid
GalA	d-galacturonic acid
UDP-GlcA	UDP-d-glucuronic acid
UDP-Glc	UDP-d-glucose
UDP-GalA	UDP-d-galacturonic acid
SDR	short-chain dehydrogenase/reductase
WSP	water-soluble pectin
