# Supplementary material for: Genome-Wide Identification and Expression Analysis of the UGlcAE Gene Family in Tomato
Source: Int J Mol Sci. 2018 May 27;19(6):1583. doi: 10.3390/ijms19061583 (PMC6032376; doi:10.3390/ijms19061583)
Supplement: Supplementary file 1 [file ijms-19-01583-s001.pdf]

Figure S1 The predicted transmembrane domains of nine SIUGlcAE proteins

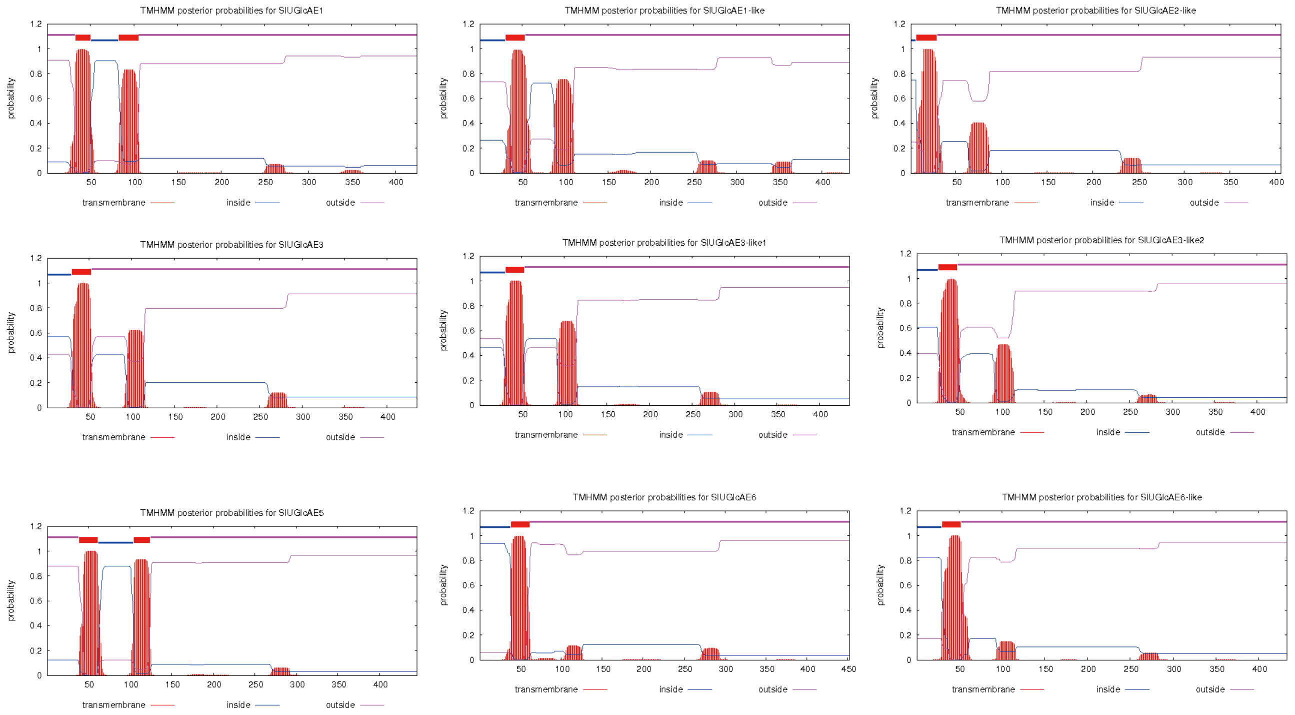

Figure S2 Selective pressure analyses of the other 8 SlUGlcAE proteins

SlUGlcAE1

Selecton Results

1	11	21	31	41
MPSLEEELFP	STPGKFKDRN	RHFHRCFAST	SNMFLWALFL	IALTASYLCF
51	61	71	81	91
QSFMDSGNRY	FTSTWGGHHW	ERNVINSAQI	RRSHGGMSVL	VTGAAGFVGS
101	111	121	131	141
HVSLALKKRG	DGVVGIDNFN	NYYDPSLKKA	RKNLLTQNSI	FIVDGDINDS
151	161	171	181	191
KLLVKLFDIA	RFTHVMHLAA	QAGVRYAMEN	PGSYIHSNIA	GLVSLLEVCK
201	211	221	231	241
NSNPQPAIVW	ASSSSVYGLN	EKVPFSESDR	TDQPASLYAA	TKKAGEEITH
251	261	271	281	291
TYNHIYGLSI	TGLRFFTVYG	PWGRPDMAYF	SFTRNILQGK	PITVYRGKNR
301	311	321	331	341
VDLARDFTYI	DDVVKGCIGS	LDTSGKSTGS	GGKKRGPAPY	RIFNLGNTSP
351	361	371	381	391
VTVPMMVAML	EKHLKVKAKK	NFVVMPGNGD	VPFTHANISS	ARKEFGYKPT
401	411	421		
TDLQTGLKKF	VKWYLSYYGY	DQGKL		

Legend:

The selection scale:

1	2	3	4	5	6	7

Positive selection — Purifying selection

SlUGlcAE1-like

Selecton Results

1 MRGLEEEFIP 11 STPGKFKDKT 21 YYSGNRQFHR 31 CFASTSTMFL 41 WALFLLALTA
51 SYLSFQSFIN 61 SGTRYLSSTW 71 GSLHWEKQVR 81 DSAQIHRVNG 91 MSVLVTGAAG
101 FVGSHVSIAL 111 KKRGDGVVGI 121 DNFNNYYDPS 131 LKKARKDLLN 141 LQNVYLIEGD
151 INDVHLISKL 161 FDIVAFTHVM 171 HLAAQAGVRY 181 AMENPKSYVH 191 SNIAGLVTLL
201 EACKNANPQP 211 AIVWASSSSV 221 YGLNEKVPFS 231 ESDRTDQPAS 241 LYAATKKAGE
251 EITHTYNHIY 261 GLSITGLRFF 271 TVYGPWGRPD 281 MAYFSFTRNI 291 LRGKPITVYR
301 GKNRVDLARD 311 FTYIDDIVKG 321 CVGSLDTAGK 331 STGSGGKKRG 341 PAMFRIFNLG
351 NTSPVTVPMM 361 VAMLEKHLKV 371 KAKKHVLDMP 381 GNGDVPFTHA 391 NISLAQKELG
401 YKPTTNLQTG 411 LGKFVRWYLS 421 YYGYNQEKFI 431 KD

Legend:

The selection scale:

1	2	3	4	5	6	7

Positive selection — Purifying selection

SlUGlcAE2-like

Selecton Results

1	11	21	31	41
MDKHRRWTYS	ITKLVFWATI	FAGAFLFFCF	RSSPPSHSTT	MKWGSSSNTP
51	61	71	81	91
QWEKRVRLSA	RSRSGHLSVL	VTGAAGFVGS	HVSAALKRRG	DGVVGLDNFN
101	111	121	131	141
SYYDPSLKRA	RQKLLEQKGV	FVMEGDINDE	KLLKKLFDIV	EFTHVMHLAA
151	161	171	181	191
QAGVRYAMKN	PGSYIHSNIA	GLVTLFEACK	SANPQPSIVW	ASSSSVYGLN
201	211	221	231	241
SKVPFSEKDQ	TDQPASLYAA	TKKAGEEIAH	TYNHIYGLSI	TGLRFFTVYG
251	261	271	281	291
PWGRPDMAYF	FFTKNILKGK	PISVFQGSNN	KSVARDFTYI	DDIVKGCLGA
301	311	321	331	341
LDTAEKSTGS	GGKKKKNAQL	RVFNLGNTSP	VPVTKLVSIL	EKLLKVKAKR
351	361	371	381	391
NVLPLPTNGD	VMFTHANISY	AHKEFGYKPT	TDLQTGLHKF	VNWYLDYYSV
401				
SEKKIY				

Legend:

The selection scale:

1	2	3	4	5	6	7

Positive selection   Purifying selection

SlUGlcAE3

Selecton Results

1	11	21	31	41
MSQMMHIDNI	PSTPGKFKME	KSPYNRLRMH	FSLAKLTFWS	FVFLGLIFVF
51	61	71	81	91
FYRSPASSSP	VSSDLSRRSL	RTSSYGGPAW	EKRIKASAKV	RSRNGISVLV
101	111	121	131	141
TGAAGFVGTH	VSVALKRRGD	GVLGLDNFND	YYDPSLKRAR	QALLERTGVY
151	161	171	181	191
VVEGDINDAT	LLKKLFDIVP	FTHVMHLAAQ	AGVRYAMENP	GSYVHSNIAG
201	211	221	231	241
LVNVLEICKS	VNPQPAIVWA	SSSSVYGLNT	KVPFSEKDRT	DQPASLYAAT
251	261	271	281	291
KKAGEEIAHT	YNHIYGLSLT	GLRFFTVYGP	WGRPDMAYFF	FTRDILKGKS
301	311	321	331	341
IPIFEAANHG	TVARDFTYID	DIVKGCLAAL	DTAEKSTGSG	GKKKGAAQLR
351	361	371	381	391
VFNLGNTSPV	PVSDLVGILE	RLLKVKAKRL	VMKLPRNGDV	PFTHANISSA
401	411	421	431	
HKELGYKPTT	DLQTGLKKFV	RWYLNYYGNG	KKSAQ	

Legend:

The selection scale:

1	2	3	4	5	6	7

Positive selection — Purifying selection

SlUGlcAE3-like2

Selecton Results

Position	Sequence
1	MSSMKHVDNI PSTPGKFKEK SHYNRLRLHF SVAKLTFWSF VFLGLIFICF
51	FKSTSSSSSP VSSDLSRRSL RTSSYDGPAW EKRIKASAKI RSTNGISVLV
101	TGAAGFVGTH VSSALKRRGD GVVGLDNFND YYDPSLKRAR QTLLESAGVY
151	IVEGDINDVA LLKKLFDIVQ FSHVMHLAAQ AGVRYAMENP SSYVHSNIAG
201	LVNLLEFCKN ANPQPAIVWA SSSSVYGLNT KVPFSESDRT DQPASLYAAT
251	KKAGEEIAHT YNHIYGLSLT GLRFFTVYGP WGRPDMAYFF FTRDMLKGKS
301	ISIFEGADHG TVARDFTYID DIVKGCLGAL DTAEKSTGSG GKKKGPAQLR
351	VFNLGNTSPV PVSDLVTILE RLLKVKAKRA VMKLPRNGDV QFTHANISLA
401	QRELGYKPTT DLQTGLKKFV RWYLSYYGEG KKSAQ

Legend:

The selection scale:

1	2	3	4	5	6	7

Positive selection — Purifying selection

SlUGlcAE5

Selecton Results

1	11	21	31	41
MTQLKPILMH	LDSIPSTPGK	FKPDKSSPYN	LYRLRFHPTL	FPRFTLWSFF
51	61	71	81	91
FIFFIVLLIF	FSSPSNPTAG	NSRRSLKNSL	SPSPALGPNW	ERRVRASARP
101	111	121	131	141
RSKTGFTVLV	TGAAGFVGTH	VSLGLKRRGD	GVLGLDNFNQ	YYDVGLKKAR
151	161	171	181	191
QSLLERSGIM	VVKGDINDAV	LLRKLFDAVA	FTHVMHMAAQ	AGVRYAMQNP
201	211	221	231	241
GSYVHSNIAG	FVSLLEACKM	ANPQPSIVWA	SSSSVYGLNS	KVPFSEKDRT
251	261	271	281	291
DQPASLYAAT	KKAGEEIAHT	YNHIYGLSIT	GLRFFTVYGP	WGRPDMAYFF
301	311	321	331	341
FTKDILRGKE	IKIFETSDQG	SVARDFTYVD	DVVKGCLGAL	DTAKKSTGSG
351	361	371	381	391
GKKKGAAQLR	IFNLGNTKPV	PVGRLVSILE	KLLKVKAKKK	VIQMPRNGDV
401	411	421	431	441
PFTHANITLA	HTELGYKPTT	DLEMGLKKFV	KWYVSYYGSK	KKSSW

Legend:

The selection scale:

1	2	3	4	5	6	7

Positive selection — Purifying selection

SlUGlcAE6

Selecton Results

1	11	21	31	41
MKTIMASPPD	TSKTTKLERY	NSYIRRVNST	KLIAASSKLL	FRVTLLVALL
51	61	71	81	91
LIFFFTINYP	PLTSEKSFNN	NIHTTTHNLL	SSAIYGGGAS	WEKQVRHSST
101	111	121	131	141
PHRPNGLSVL	VTGAAGFVGS	HCSLALKKRG	DGVLGLDNFN	SYYDPSLKRA
151	161	171	181	191
RQDQLSKHQI	FIVEGDINDT	ELLKKLFDIV	PFTHILHLAA	QAGVRYAMQN
201	211	221	231	241
PLSYVNSNVA	GFVNLLEIAK	AADPQPAIVW	ASSSSVYGLN	TKVPFSEDHR
251	261	271	281	291
TDQPASLYAA	TKKAGEAIAH	TYNHIYGLSL	TGLRFFTVYG	PWGRPDMAYF
301	311	321	331	341
FFTKDMIQGK	SINVYVTQDD	KEVARDFTYI	DDIVKGCVGA	LDTAEKSTGS
351	361	371	381	391
GGKKRGPAQL	RVYNLGNTSP	VSVKKLVAIL	ENLLNLKAKK	FFIKMPRNGD
401	411	421	431	441
VPFTHANVSL	ALRDFGYKPT	TDLSSGLRKF	VKWYVSYYGI	QSRVKKESEG
451				
EK				

Legend:

The selection scale:

1	2	3	4	5	6	7

Positive selection   Purifying selection

SlUGlcAE6-like

Selecton Results

```
1          11         21         31         41
MASLFSIDTK KSIKLDRNTY IHKVNSSTKL IVAFIKLFYC IAILISALLI
51         61         71         81         91
GLFILNHILF ISTDSNQHGR RLFSFGGGAG WEKKVRHSSI PKKINGKVVL
101        111        121        131        141
VTGAAGFVGS HCGLALKKRG DGVIGIDNFN SYYDPSLKRA RQELMSKHEI
151        161        171        181        191
FIVDGDINDI DLVNKLFDIV RFTHVLHLAA QAGVRYAMEN PLSYVHSNVN
201        211        221        231        241
GFVNLLEIAK SANPQPAIVW ASSSSVYGLN TKVPFSENDR TDQPASLYAA
251        261        271        281        291
TKKAGEEIAH TYNHIYGLSL TGLRFFTVYG PWGRPDMAYF FFTKDMIQGK
301        311        321        331        341
PIKVYVSDKE VARDFTYIDD VVKGCLGAID TAEKSTGSGG KKRGPAQLRV
351        361        371        381        391
YNLGNTSPVS VKKLVAILEN LLNIKAKKNL IRMPRNGDVP FTHANVSLAY
401        411        421        431
RDFGYKPTTN LSSGLRKFVK WYLSYYGIQA RIK
```

Legend:

The selection scale:

1	2	3	4	5	6	7

Positive selection Purifying selection

Figure S3 Selective pressure in promoter regions of *SlUGlcAE* genes

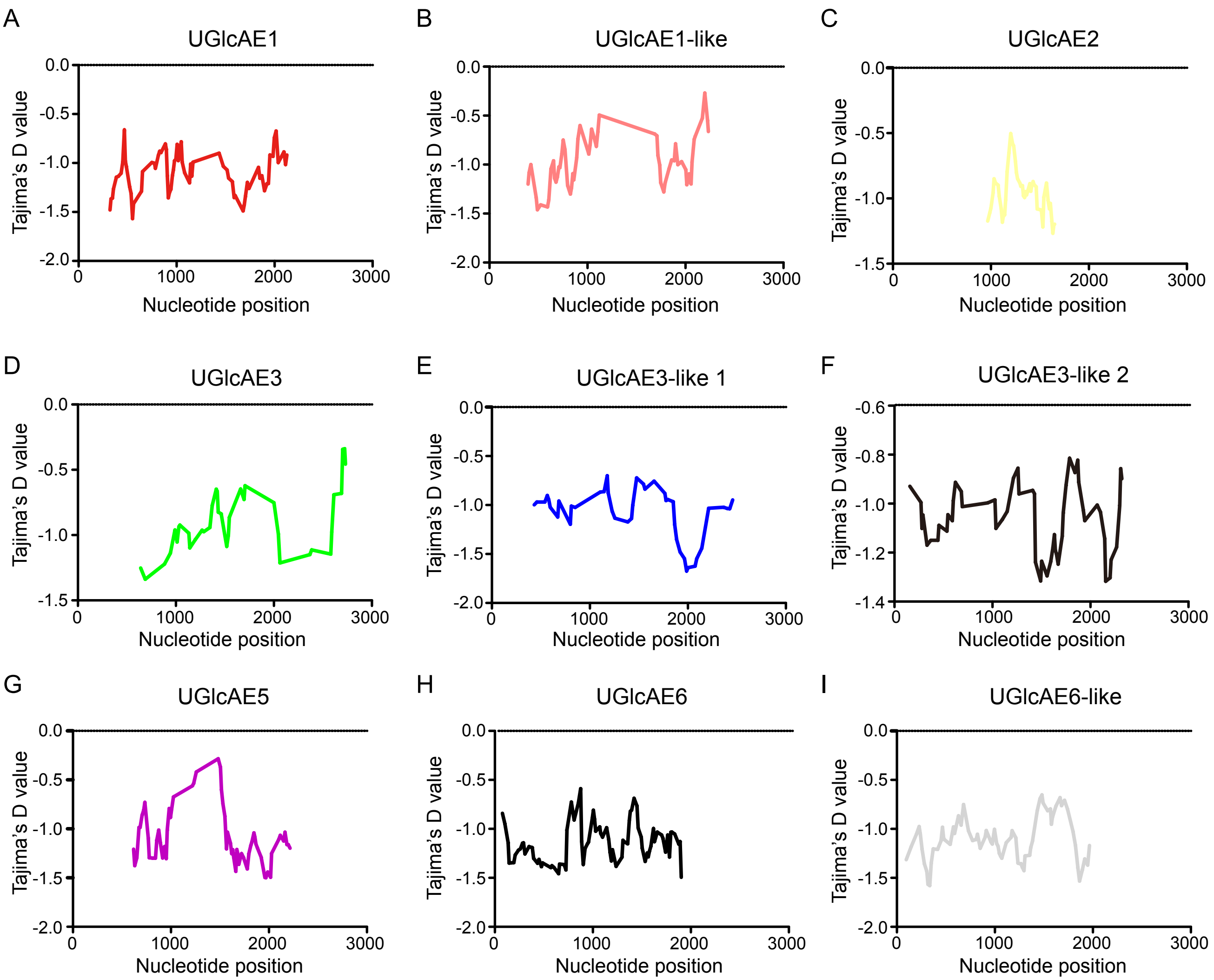

Table S1 Kinds and numbers of *cis*-acting elements in the 0.5 kb upstream regions of 9 *SlUGlcAE* genes

Gene sample	HSE	MBS	Skn-1_motif	TGA-element	HD-Zip 1	HD-Zip 2	TC-rich repeats	circadian	CAT-box	TATC-box	W box	WUN-motif	LTR	TCA-element	ELI-box3
SlUGlcAE1	1	1	1	1	0	0	0	0	0	0	0	0	0	0	0
SlUGlcAE1-like	0	0	0	0	1	1	1	1	0	0	0	0	0	0	0
SlUGlcAE2-like	0	0	0	0	0	0	0	0	1	1	1	0	0	0	0
SlUGlcAE3	1	0	0	0	0	0	0	0	0	0	0	0	0	0	0
SlUGlcAE3-like1	0	0	0	0	0	0	1	1	0	0	0	0	0	0	0
SlUGlcAE3-like2	0	0	0	0	0	0	1	2	0	0	0	1	0	0	0
SlUGlcAE5	1	0	0	0	0	0	1	0	0	0	0	0	1	2	0
SlUGlcAE6	2	0	1	0	0	0	0	0	0	0	0	0	0	0	1
SlUGlcAE6-like	0	0	0	0	0	0	0	0	0	0	0	0	0	0	0

Table S2 Kinds and numbers of cis-acting elements in the 1.5 kb upstream regions of 9 *SlUGlcAE* genes

Gene sample	ABRE	ARE	HSE	MBS	TGA-element	TGACG-motif	CGTCA-motif	TC-rich repeats	circadian	AuxRR-core	GARE-motif	LTR	TATC-box	P-box	ERE	WUN-motif	MRE	TCA-element	HD-Zip 1	HD-Zip 2	Skn-1_motif	GCN4_motif	O2-site	as-2-box	W box	CAT-box	GC-motif	CCGTCC-box	ELI-box3
SlUGlcAE1	2	1	1	1	1	2	0	0	0	0	0	0	0	0	0	0	0	0	1	1	1	0	0	0	0	0	0	0	0
SlUGlcAE1-like	0	0	0	0	0	1	1	1	1	0	0	0	0	0	0	0	0	0	1	1	1	1	1	1	0	0	0	0	0
SlUGlcAE2-like	0	0	0	0	0	1	0	0	1	1	1	1	1	0	0	0	0	0	0	0	1	2	0	0	1	2	1	0	0
SlUGlcAE3	0	0	1	0	0	0	0	1	0	0	0	0	0	1	0	0	0	0	0	0	0	0	0	0	0	0	0	0	0
SlUGlcAE3-like1	0	0	0	0	0	0	0	1	2	0	0	0	0	0	0	0	0	0	0	0	1	0	0	0	0	0	0	0	0
SlUGlcAE3-like2	0	0	2	1	0	0	0	2	2	0	0	0	0	0	1	1	0	0	0	0	0	0	0	1	1	0	0	1	0
SlUGlcAE5	0	0	1	0	0	0	0	3	2	0	0	1	0	0	1	0	1	2	0	0	0	0	0	0	0	0	0	0	0
SlUGlcAE6	0	2	2	1	0	0	0	1	0	0	0	0	0	0	1	0	0	0	0	0	2	1	0	0	1	0	0	0	1
SlUGlcAE6-like	0	0	2	0	0	0	0	1	0	0	0	0	0	0	0	1	0	0	0	0	0	0	0	0	0	0	0	0	0
